# Serum free light chain level-based and non-fixed cycle daratumumab treatment strategy for patients with light chain amyloidosis

**DOI:** 10.1080/07853890.2024.2442075

**Published:** 2024-12-19

**Authors:** Zhen Li, Jinzhou Guo, Wencui Chen, Liang Zhao, Guisheng Ren, Xianghua Huang

**Affiliations:** National Clinical Research Center for Kidney Diseases, Jinling Hospital, Affiliated Hospital of Medical School, Nanjing University, Nanjing, China

**Keywords:** AL amyloidosis, daratumumab, non-fixed cycle therapy

## Abstract

**Background:**

In recent years, daratumumab (DARA) has gained widespread use in the treatment of systemic light chain (AL) amyloidosis. In this study, we assessed the efficacy and safety of a DARA treatment strategy based on serum free light chain (sFLC) levels and non-fixed cycles.

**Methods:**

The study included 123 patients with Al amyloidosis who received DARA at our center between July 2020 and September 2023. All patients received the standard DARA treatment (16 mg/kg weekly for four weeks) during the first course. Subsequent treatments were adjusted based on sFLC levels and the physician’s judgment.

**Results:**

The results demonstrated an impressive overall hematologic response rate (ORR) of 94.3%, with a hematologic very good partial response (VGPR) and complete response (CR) rate of 84.5%. Median time to best hematologic response was 1 months. Cardiac and renal response rates were 39.3% and 60.3%, respectively. Thirty patients experienced grade 1/2 infusion-related reactions after the first infusion. The rate of grade 3/4 adverse events was 21%. The most common adverse events of grade 3 or 4 were pulmonary infection (6.5%), neutropenia and lymphocytopenia (5.7%), elevated transaminases (1.6%), acute kidney injury (1.6%). After a median follow-up of 13 months (range 1-38), The 1-year OS and PFS estimates were 96.5% and 84.4%, respectively.

**Conclusion:**

These findings indicate that the sFLC levels based and non-fixed cycle DARA strategy is an efficacious and safe treatment strategy in both newly diagnosed and relapsed/refractory AL amyloidosis.

## 1. Introduction

Systemic immunoglobulin light chain (AL) amyloidosis is caused by the folding of light chains produced by abnormal plasma cells into highly organized amyloid fibrils that deposit in tissues, resulting in progressive organ damage and dysfunction [1]. The clinical manifestations of AL amyloidosis are diverse, mainly involving the heart and kidneys. The prognosis of this disease depends mainly on the degree of organ dysfunction [2]. Over the past 20 years, outcomes for patients with AL amyloidosis have improved with timely diagnosis, use of chemotherapeutic agents, or autologous hematopoietic stem cell transplantation (ASCT) [3]. However, some patients with advanced disease at the time of initial presentation were not candidates for cytotoxic chemotherapy or ASCT, and the complete response (CR) and organ response rates of the previous regimen were still unsatisfactory. Daratumumab (DARA) represents a breakthrough in the treatment of AL amyloidosis in recent years [4]. Based on the results of the ANDROMEDA trial, the Food and Drug Administration (FDA) has approved DARA in combination with CyBorD (bortezomib + dexamethasone + cyclophosphamide) for the treatment of newly diagnosed AL amyloidosis. A series of retrospective studies have also demonstrated impressive rapid and profound hematologic response outcomes and prolonged overall survival with DARA treatment under fixed cycles in patients with AL amyloidosis [5–8], but the dosage and duration of DARA treatment are derived from multiple myeloma, and the optimal treatment plan in patients with AL amyloidosis still needs further exploration. Based on previous experience with bortezomib or ASCT for the treatment of patients with AL amyloidosis, some patients can achieve a long-term response from short-term treatment, and many patients with AL amyloidosis receiving DARA treatment have a very rapid onset of effect [9–11]. Therefore, to save medical resources and reduce the complications of long-term DARA treatment for patients, we launched this study and aimed to investigate the efficacy of serum free light chain (sFLC) level-based and non-fixed cycle DARA treatment plans for patients with AL amyloidosis.

## 2. Patients and Methods

### 2.1. Patients and treatment plan

In this retrospective study, we enrolled 123 consecutive patients with histologically diagnosed AL amyloidosis who received DARA-based treatment at the National Kidney Disease Clinical Research Center of Jinling Hospital from July 2020 to September 2023. Among these patients, 41 patients were newly diagnosed and the other 82 patients were relapsed or refractory patients. The diagnosis of AL amyloidosis in patients was established through a pathological diagnosis that included positive Congo red staining confirmed by pathological biopsy, and κ or λ light chain confirmation by immunofluorescence. Patients with localized, hereditary or secondary amyloidosis were excluded. The study was approved by the institutional review boards or ethics committees of Jinling Hospital and was conducted according to the Declaration of Helsinki, the International Conference on Harmonization, and the Guidelines for Good Clinical Practice. Written informed consent to participate in the study was obtained from all patients.

The sFLC level-based and non-fixed cycle DARA treatment plan was as follows: IV DARA at 16 mg/kg weekly for 4 doses and dexamethasone at 20-40 mg with DARA infusions. Subcutaneous bortezomib was administered 1.3 mg/m^2^ on days 1, 8, 15, and 22 every 4 weeks if needed. After the first course of treatment, all patients underwent a hematological response assessment. If the patient achieved a hematological CR, subsequent treatment was carried out according to the physician’s judgment and sFLC level. Patients with other hematological responses continued to receive standard therapy until they reached hematological CR. Patients who did not achieve VGPR after 2 courses of treatment were adjusted to other treatment plans. Primary endpoints include hematologic response, organ response, and safety, with secondary endpoints of progression-free survival (PFS) and overall survival (OS).

### 2.2. Related standards and definitions

Criteria for assessing organ involvement refer to the Consensus Opinion from the 10th International Symposium on Amyloid and Amyloidosis [12]. Renal involvement in AL amyloidosis was defined as non–Bence Jones proteinuria of more than 0.5 g/d. Cardiac involvement defined as cardiac ultrasound mean ventricular wall thickness >12 mm, excluding other cardiac diseases; or N-terminal natriuretic peptide type B (NT-pro BNP) >332 ng/L in the absence of renal insufficiency and atrial fibrillation, and staging patients with AL amyloidosis using the Mayo 2004 and Mayo 2012 staging systems [13,14]. Relapse is defined as hematologic and/or organ progression after 3 months of response with treatment and maintenance, while refractory is defined as no hematologic or organ response after 3 months of treatment with the initial regimen. For the assessment of hematologic response [15], complete response (CR) was defined in patients with the difference between serum involved and uninvolved free light chain (dFLC) > 50 mg/L if the blood free light chains were completely normal and serum and urine immunofixation electrophoresis were negative after treatment; very good partial response (VGPR) was defined if serum dFLC was < 40 mg/L; partial response (PR) was defined if serum dFLC decreased by more than 50%; and hematological nonresponse (NR) was defined if PR was not achieved. In patients with baseline serum dFLC in the range of 20-50 mg/L, if dFLC was < 10 mg/L after treatment, it was defined as a PR, and the opposite was considered NR. The hematological overall response rate (ORR) was defined as PR or better. The criteria for renal response were a 50% decrease in urine protein quantification (at least ≥ 0.5 g/d) and a rise in serum creatinine of no more than 25% of baseline [16], while the criteria for cardiac response were: a decrease of > 30% of baseline for those with baseline NT-pro BNP > 650 ng/L or more and a decrease of 300 ng/L or more [17]. Progression-free survival (PFS) was defined as the time from the start of DARA to the date of progression, death, or last follow-up; overall survival (OS) was defined as the time from the start of DARA to the date of death or last follow-up. Adverse events (AEs) were assessed using the Common Terminology Criteria for AEs version 5.0 (CTCAE v5.0).

### 2.3. Statistical methods

The SPSS 25. 0 software was used for statistical purposes. Normally distributed measures are described as the mean ± standard deviation, and independent samples t-tests were used for comparisons between groups. Nonnormally distributed measures are described as the median (interquartile spacing), and the Mann-Whitney U test was used for comparisons between groups. Categorical variables are expressed as absolute numbers (rates), and the χ2 test or Fisher’s exact test was used to compare the count data between groups. Survival probabilities were estimated using the Kaplan-Meier method, and log-rank tests were used for comparisons between groups. All tests were two-sided, and *p* < 0.05 was considered statistically significant.

## 3. Results

### 3.1. Baseline characteristics

A total of 123 patients with AL amyloidosis (41 newly diagnosed and 82 relapsed or refractory patients, respectively) were included in this study, with a median age of 59 (range 35-78) years and 63 (51.2%) males. 106 cases (86.2%) were λ-type and 17 cases (13.8%) were ĸ-type. Seventy-four (60.2%) patients had cardiac involvement, 123 (100%) patients had renal involvement, and 75.6% had involvement of 2 or more organs. The majority of patients (58.6%) were classified as having a cardiac stage of II or higher. The median percentage of bone marrow plasma cells was 3.5% (range 0.4-19), and the median value of urine protein was 4.14 g/24h (range 0.2-15.97). The median baseline NT-pro BNP in patients with cardiac involvement was 1481.1 ng/L (range 124.1-35105). The median time from diagnosis to receiving DARA in the newly diagnosed and relapsed or refractory groups was 3 days (range 1-38) and 30 days (range 15-510), respectively. One hundred and four (84.6%) patients received DARA and dexamethasone (DD) and 19 (15.4%) patients received DARA + bortezomib + dexamethasone (DVD). The median number of lines of therapy was 3 (2-5) for relapsed or refractory patients. Seventy-four patients (60.2%) were previously treated with bortezomib, 37 (30.1%) were previously treated with immunomodulatory inhibitors (IMiDs), and 24 (19.5%) had ASCT. Patient baseline information is detailed in Table 1.

**Table 1.** Characteristics of the study population.

Clinical characteristic	All patients (*n* = 123)	Newly diagnosed (*n* = 41)	Relapse/Refractory (*n* = 82)	P - Value
Age at initiation of DARA, years Median (range)	59(35-78)	59 (35-77)	60 (40-78)	0.727
Gender, male/female, n (%)	63/60(51.2/48.8)	22/19(53.7/46.3)	41/41(50/50)	0.702
Isotype, lambda/kappa, n (%)	106/17(86.2/13.8)	35/6(85.4/14.6)	71/11(86.6/13.4)	0.724
Time from diagnosis to initiation of DARA, days, Median (range)	15(1-510)	3 (1-38)	30 (15-510)	**<0.001**
Treatment line of DARA, n (%)				
2^nd^	35 (28.4)		35 (42.7)	
3^rd^	31 (25.2)	/	31 (37.8)	/
> 3^rd^	16 (13)		16 (19.5)	
Prior treatment, n (%)				
PI	74 (60.2)	/	74 (90.2)	/
IMiD	37 (30.1)		37 (45.1)	
ASCT	24 (19.5)		24 (29.2)	
Number of DARA treatments, Median (range)	5(3-18)	5 (3-18)	5 (3-16)	0.272
Lines of prior treatments, Median (range)	2(1-5)	/	3(2-5)	/
Cytogenetic abnormality, n (%)	15/27 (55.6)	3/9 (33.3)	12/18 (66.7)	0.218
Translocation (11; 14), n (%)	8/27 (29.6)	1/9 (11.1)	7/18 (38.9)	0.297
Organ involvement, n (%)				
Renal	123(100)	41(100)	82(100)	/
Cardiac	74 (60.2)	22 (53.7)	52 (63.4)	0.297
Hepatic	5 (4.1)	2 (4.9)	3 (3.7)	1
Nervous system	2 (3.3)	1 (2.4)	3 (3.7)	1
Gastrointestinal tract	23 (18.7)	6 (14.6)	17 (20.7)	0.414
Soft tissue	53 (43.1)	17 (41.5)	36 (43.9)	0.797
Mayo stage 2004, I/II/III, n (%)	51/29/43 (41.5/23.6/35)	20/4/17 (48.8/9.8/41.5)	31/25/26 (37.8/30.5/31.7)	**0.035**
Mayo 2012 Stage, I/II/III/IV, n (%)	48/39/29/7 (39/31.7/23.6/5.7)	13/11/12/5 (31.7/26.8/29.3/12.2)	35/28/17/2 (42.7/34.1/20.7/2.4)	0.101
NYHA, I/II/III/IV, n (%)	67/42/10/4 (54.4/34.1/8.1/3.3)	23/12/5/1 (56.1/29.3/12.2/2.4)	44/30/5/3 (53.7/36.6/6.1/3.7)	0.62
Renal Stage, I/II/III, n (%)	57/48/17 (46.7/39.3/13.9)	22/16/2 (53.7/39/4.9)	35/32/15 (42.7/39/18.3)	0.117
ECOG PS score, Median (range)	1 (1-4)	2(1-3)	1(1-4)	0.408
0-2, n (%)	111 (90.3)	35 (85.4)	76 (92.7)	
≥ 3, n (%)	12 (9.7)	6 (14.6)	6 (7.3)	
Bone marrow plasma cell % Median (range)	3.5 (0.4-19)	3 (1-19)	3.5 (0.4-13)	0.809
NT-pro BNP with cardiac involvement (ng/L), Median (range)	1481.1 (124.1-35105)	2272.5 (298.1-35105)	1369.3 (124.1-24004)	0.222
Troponin-T, pg/mL, Median (range)	0.026 (0.003-0.275)	0.037 (0.003-0.275)	0.025 (0.004-0.197)	0.730
eGFR, mL/min/1.73m^2^, Median (range)	71 (7-123)	83 (14-122)	69 (7-123)	0.064
24-hour urine protein (g), Median (range)	4.11 (0.2-15.97)	4.09 (0.52-11.2)	4.36 (0.23-15.97)	0.056
dFLC (mg/L), Median (range)	87.5 (3.7-2382.8)	163.9 (9.9-2382.8)	58.1 (3.7-832)	**<0.001**
iFLC (mg/L), Median (range)	111 (15.1-2397.5)	173 (18-2397.5)	89.8 (15.1-855)	**<0.001**
LVEF, % Median (range)	60 (31-79)	61 (31-79)	60 (33-78)	0.727

ASCT: autologous stem cell transplantation; DARA: daratumumab; dFLC: difference in free light chains; ECOG PS: Eastern Cooperative Oncology Group Performance Status; eGFR: estimated glomerular filtration rate; iFLC: involved free light chain; IMiD: Immunomodulatory Drug; LVEF: left ventricular ejection fraction; NT-proBNP: N-terminal natriuretic peptide type B; NYHA: New York Heart Association; PI: Proteasome Inhibitor.

### 3.2. Response evaluation: hematological and organ response

**Figure 1.**
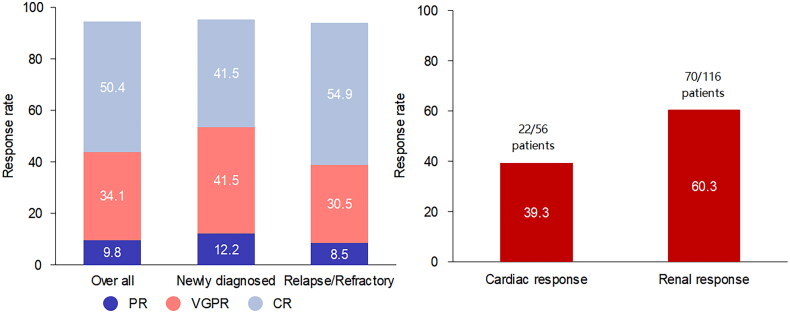
Best hematological response and organ response in all patients.

**Figure 2.**
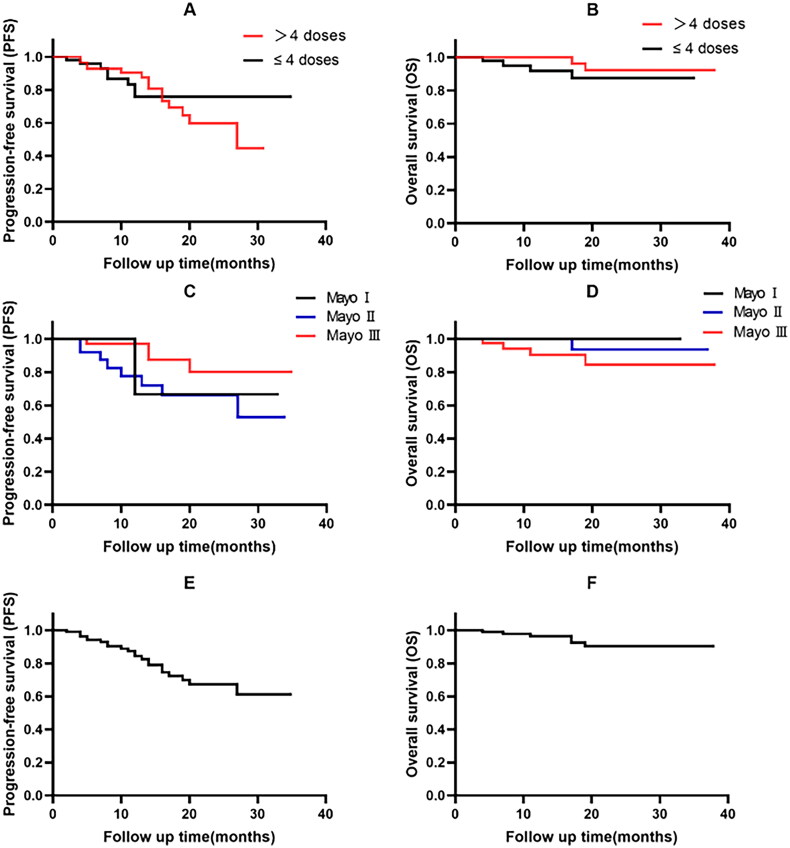
Overall and progression-free survivals. (A) Progression-free survival and number of treatments. (B) Overall survival and number of treatments. (C) Progression-free survival and Mayo stage. (D) Overall survival and Mayo stage. (E) Progression-free survival in all patients. (F) Overall survival in all patients.

By March 2023, 123 patients received a median of 5 (range 3-18) doses of DARA treatments. The median duration of DARA treatment was 3 months (range 1-38). Hematologic response was achieved in 116 patients, with a hematologic ORR of 94.3%. Among the hematologic response patients, 62 (50.4%) achieved hematologic CR, 42 (34.1%) achieved hematologic VGPR, and 12 (9.8%) patients achieved hematologic PR (Figure 1). Median time to best hematologic response was 1 months (range 0.5-27). There was no significant difference in hematologic response between newly diagnosed patients and relapsed or refractory patients (95.1% vs. 93.9%, *p* = 1.0). Fifty-nine patients received ≤ 4 doses of DARA treatments and a total of 55 patients achieved hematologic response (with a hematologic ORR of 93.2%) of whom 26 (44.1%) achieved hematologic CR and 22 (37.3%) achieved hematologic VGPR. There was no statistically significant difference in OS and PFS between the number of DARA treatments > 4 doses and ≤ 4 doses. (*p* = 0.147, *p* = 0.166) (Figure 2A,B).

Of the 74 patients with cardiac involvement, 18 patients could not be evaluated because of baseline NT-pro BNP levels < 650 ng/L or lack of follow-up data. Twenty-two of 56 evaluable patients (39.3%) had a cardiac response, 3 (5.4%) achieved cardiac CR, 5 (8.9%) achieved cardiac VGPR, and 14 (25%) had cardiac PR. At 6 months after treatment (*n* = 34), 14 (CR for 2, VGPR for 5, PR for 7) patients achieved cardiac responses, and 20 patients did not have a response. At 6-month landmark analysis, patients who achieved a deep cardiac response had a higher 1-year survival rate (100% vs. 94.4%, *p* = 0.556). Of the 123 patients with renal involvement, 116 were evaluated for renal response, and 70 (60.3%) achieved renal response, including 34 (29.3%) with renal VGPR or better and 36 (31%) with renal PR. At 6 months after treatment (*n* = 83), 44 (CR for 1, VGPR for 17, PR for 26) patients achieved renal response, and 39 patients had no responses. The median time to optimal cardiac response and renal response after treatment was 3.5 (range 1-19) and 4 (range 1-36) months, respectively. Data regarding hematologic responses and organ responses are shown in Figure 1 and Table 2.

**Table 2.** Organ response.

		N	CR	VGPR	PR	NR
Cardiac response	Newly diagnosed	16/22	1/16	2/16	3/16	10/16
	Relapse/Refractory	40/52	2/40	3/40	11/40	24/40
Renal response	Newly diagnosed	39/41	0	13/39	15/39	11/39
	Relapse/Refractory	77/82	3/77	18/77	21/77	35/77

CR: complete response; VGPR: very good partial response; PR: partial response; NR: non response.

After one course of DARA treatment, 44 patients (35.7%) achieved hematologic CR and were then judged to continue treatment based on clinician experience. The remaining 79 patients continued DARA fixed cycle treatment until they achieved hematologic CR. The median number of DARA treatments was 4 (3-13) and 5 (3-18), respectively (*p* = 0.097). Hematologic ORR was 100% and 91%, respectively (*p* = 0.104). In patients who can be evaluated, cardiac response rates were 45% and 36%, and renal response rates were 60.4% and 60.2%, respectively (*p* = 0.514, *p* = 0.586). There was no difference in the median time to best hematologic and optimal cardiac and renal response between patients with CR and those without CR after the first course of treatment (*p* = 0.092, *p* = 0.072, *p* = 0.062). The treatment results of these two groups are shown in Table 3.

**Table 3.** Treatment results.

	CR (*n* = 44)	No-CR (*n* = 79)	P-Value
Number of DARA treatments, Median (range)	4(3-13)	5(3-18)	0.097
Median time to best haematologic response (range)	1 (0.5-22)	2 (0.5-27)	0.092
Overall cardiac response, n/N (%)	9/20(45%)	13/36(36%)	0.514
Median time to cardiac response in months (range)	12 (1-16)	3 (1-19)	0.072
Overall renal response, n/N (%)	26/43 (60.4%)	44/73 (60.2%)	0.586
Median time to renal response in months (range)	5 (1-23)	3 (1-36)	0.062

CR: complete response; DARA: daratumumab.

Forty-three patients with advanced cardiac involvement were enrolled in this study, and 93% (40 patients) are still alive. The hematologic ORR was 93% (51.2% CR, 27.9% VGPR, 14% PR). Fourteen of 37 evaluable patients (37.8%) had a cardiac response. Hematologic responses were similar in patients IIIa and IIIb (OR 34/37 and 6/6, respectively, and CR 3/6 and 19/37, respectively), 11/31 patients with stage IIIa AL had a cardiac response, while 3/6 patients with stage IIIb had a similar response. Median time to cardiac response was 3 months after treatment. Median OS and PFS were not reached. There was no statistically significant difference in OS and PFS between the number of Mayo I, II and Mayo III. (*p* = 0.141, *p* = 0.1) (Figure 2C,D).

### 3.3. Adverse events

The most common adverse event in the study was grade 1/2 infusion-related reactions (IRRs) after the first infusion, which was observed in 30 patients (24.4%). Most patients presented with cough, asthma, chest distress, and throat discomfort, which mostly occurred within 1-3 h after the start of infusion. After slowing down of the infusion rate or suspension of the infusion, the patients could experience spontaneous relief without the need to terminate the treatment. One patient developed anaphylactic shock within half an hour after DARA infusion. The patient improved after symptomatic treatment and continued to complete DARA therapy. Other non-infusion related adverse reactions included fever (5 patients), constipation (6 patients), diarrhea (1 patient), abdominal distention (4 patients), and muscle discomfort (2 patients), which improved after symptomatic treatment. The rate of grade 3/4 adverse events was 21%. The most common adverse events of grade 3 or 4 were pulmonary infection (6.5%), neutropenia and lymphocytopenia (5.7%), elevated transaminases (1.6%), acute kidney injury (1.6%). No grade 5 adverse events occurred. One patient had recurrent viral pneumonia after the first infusion, which was considered to be related to DARA infusion toxicity, and hematological CR was achieved by completing 1 course of DARA after 2 years of treatment suspension. The most common any-grade and grade 3/4 AEs are reported in Table 4.

**Table 4.** Most common adverse events during treatment.

Events	Patients (*n* = 123)	
	All grade AEs N (%)	Grade 3/4 AEs N (%)
Infusion-related reactions	30(24.4)	0
Fever	5(4.1)	0
Constipation	6(4.9)	1(0.8)
Diarrhea	1(0.8)	0
Distention	4(3.3)	1(0.8)
Muscle discomfort	2(1.6)	1(0.8)
Skin rash	1(0.8)	1(0.8)
Neutropenia and lymphocytopenia	7(5.7)	7(5.7)
Pulmonary infection	8(6.5)	8(6.5)
Fungal infection	1(0.8)	1(0.8)
Herpes zoster	1(0.8)	1(0.8)
Acute kidney injury	2(1.6)	2(1.6)
Elevated transaminases	3(2.4)	2(1.6)
Anaphylactic shock	1(0.8)	1(0.8)

### 3.4. Survival outcome

One hundred and seventeen of 123 patients (95.1%) are still being followed up, with a median follow-up time of 13 months (range 1-38). Six patients (4.9%) died. Four died of cardiac complications due to amyloidosis (one from cardiac arrest and three from refractory heart failure). Two died of end-stage multiple organ failure. Twenty-two patients (17.9%) who were evaluated for hematologic relapse continued DARA therapy after relapse and had good survival outcomes, with 90.1% of patients still alive. Seven patients (5.7%) showed hematologic refractory to DARA treatment, and one patient achieved hematological VGPR and underwent ASCT treatment. After one course of DARA treatment, patients achieving CR had a higher 1-year survival rate (97.1% vs. 95.9%, *p* = 0.658) compared to patients without CR. Median OS and PFS were not reached. The 1- and 2-year OS estimates were 96.5% and 90.4%, respectively, and the 1-year PFS rate was 84.4% (Figure 2E,F).

## 4. Discussion

AL amyloidosis is a complex multisystem disease that challenges physicians from diagnosis to treatment [18]. DARA, a drug used to treat AL amyloidosis, has been shown to have significant efficacy in improving symptoms and prolonging survival in patients [19]. The existing treatment plan requires patients to receive DARA treatment regularly for at least two years, which may lead to an increase in treatment-related complications and greatly increase the financial burden on patients [20]. However, whether reducing the number of treatments will lead to a decrease in treatment response rate is also the most concerning issue in clinical practice. Therefore, we attempt to answer the above questions through this retrospective cohort study.

This study evaluated the efficacy of DARA therapy based on serum free light chain levels, rather than using fixed cycle therapy. In our study, we report a hematologic ORR of 94.3%, with a hematologic VGPR or better rate of 84.5%, which is similar to other reported results [21,22]. Organ responses also benefited from early and deep hematological responses, with response rates of 39.3% and 60.3% in the cardiac and renal organs, respectively. The optimal organ response time is later than the optimal hematological response time. It is a reminder that organ responses can be delayed after achievement of a hematologic response, and, hence, additional treatment or changes in treatment should not be considered due to lack of organ response in the first 6 to 12 months, as long as hematologic responses are favorable [23]. Muchtar et al. [17] reported that depth of organ response was associated with improved survival in AL amyloidosis. However, due to the short follow-up time, we have not yet observed a relationship between 6-month organ response rate and survival in this group of patients. In Sanchorawala et al.’s study, the hematologic ORR was 90% after 24 standard DARA treatment cycles, the hematologic VGPR or better rate was 86%, and the response rates of the heart and renal organs were 50% and 67%, respectively [21]. Similarly, the fixed-cycle DARA treatment regimen achieved a hematologic ORRs ranging from 72% to 86% [5–8]. Given that DARA induced rapid and profound hematologic responses and that the depth of the response had an impact on the duration of the response, fast relapse was less likely in patients who achieved VGPR or better, normal iFLC levels, or very low dFLC (dFLC < 10 mg/L) [24]. This suggests that extending the interval between treatments is feasible. In this study, the hematologic and organ response rates of patients with AL amyloidosis treated with non-fixed cycles of therapy were comparable to other studies. In addition, there was no significant difference in the results we grouped according to whether hematologic CR was achieved after the first course of treatment. Based on the above data, it seems that our treatment regimen can achieve similar results compared standard therapy.

The non-fixed cycle DARA regimen was well tolerated. Grade 1/2 IRRs occurred in 30 (24.4%) of patients in our study, most of which were cough, asthma, chest tightness, and throat discomfort. All of them recovered spontaneously without discontinuation of treatment. In an analysis of mate that included 977 patients, 15 studies reported IRRs. Of the included 276 patients who received DARA treatment, 87 experienced grade 1 or 2 IRRs (33%), 10 of 432 patients (3%) experienced grade 3 or 4 IRRs [25]. In addition, infections are a common toxicity associated with this agent in treatment of AL amyloidosis. The incidence of infection-related complications in this study was 9/123(7.3%), and most of them were controllable. Only one patient suspended DARA therapy due to recurrent pulmonary viral infection after the first infusion. In comparison, the rate of grade 3 or 4 infections in the ANDROMEDA Phase III trial was 16.6% and the incidence of serious adverse events was 43.0%. The percentage of patients who had adverse events that led to discontinuation of trial treatment was 4.1% [26]. It seems that non-fixed cycle DARA treatment for AL amyloidosis can reduce the incidence of grade 3/4 AEs and DARA-related complications.

After a median follow-up of 13 months (range 1-38), the survival results of 22 relapsed patients were encouraging, with 90.1% currently still alive, supporting the role of this drug in relapsed or refractory patients. In a mate-analysis Jian Li et al. concluded that three studies reported a 1-year PFS of 58% [25]. In another multicenter retrospective study, 12-month OS and PFS were 59% (95%CI: 0.36–0.77) and 52% (95%CI: 0.29–0.70), respectively [8]. In this study, 1-year OS and PFS were 96.5% and 84.4%, respectively. Notably, there were no relapses or deaths in the newly diagnosed group after treatment. In comparison, 1-year OS and PFS were 95.3% and 79.1%, respectively, in the relapsed/refractory group. Compared to other studies, our study shows remarkable survival results. We also observed that patients with hematologic ≥ VGPR had a higher 1-year survival rate (1-year survival, 98.6% vs. 82.5%, *p* = 0.002). We attribute this to a deep and rapid hematological response. This further demonstrates the feasibility of reducing the number of DARA treatments.

In patients with advanced cardiac involvement, our non-fixed cycle therapy also induced a rapid and profound response. In this study, 43 patients with cardiac stage III were included, and 93% (40 patients) were still alive. The hematologic ORR was 93% and 51.2% patients achieved a CR (51.3% with stage IIIa and 50% with stage IIIb). The organ response rate was 37.8% (35.5% with stage IIIa and 50% with stage IIIb). It can be observed that the study of Roman Gounot et al. reported a hematologic ORR of 73.9%, and a hematologic CR of 34.8% (36.3% with stage IIIa and 33.3% with stage IIIb). Additionally, 30.4% of the patients showed a cardiac response, and at the time of the study reporting, 52% of the patients were still alive [27]. Bortezomib use to patients with stage III cardiac involvement was limited – it was only given to 5 patients with stage III cardiac disease – due to concern that it can exacerbate cardiac function. The decision for the selection of partner of daratumumab was per the clinician’s experience.

Our series, is constrained by its retrospective nature, which may result in incomplete data reporting, particularly in the assessment of organ response and toxicity, which may have influenced the reliability of the results. Moreover, some cases were lost to follow-up, which may have introduced selection bias. Additionally, the follow-up of our cohort is relatively short, and further observations remain to be made regarding organ response and long-term patient outcomes. Therefore, we are currently unable to determine the optimal number of treatments for DARA and cannot adequately assess the long-term efficacy and safety of the treatment regimen.

## 5. Conclusion

The data from our study support the high efficacy and safety of the DARA regimen administered under non-fixed cycles for both newly diagnosed and relapsed or refractory AL amyloidosis patients. We observed a hematological ORR of 94.3% and a response rate of 39.3% and 60.3% for cardiac and renal organs, respectively. Furthermore, our findings indicate the 1-year OS and PFS estimates were 96.5% and 84.4%, respectively. The incidence of grade 3 or 4 AEs was 21%. This personalized treatment strategy can reduce the number of treatments while maintaining hematological efficacy and reducing the incidence of severe complications. However, the optimal duration of DARA for the treatment of patients with AL amyloidosis is still unknown and we suggest that more studies should be carried out to explore the better use of DARA in patients with AL amyloidosis.

## Data Availability

Data are available upon reasonable request. All data is available upon request to the corresponding author, please contact hxhszb@163.com.
